# Impact of an antimicrobial stewardship program on optimizing linezolid consumption and susceptibility in intensive care unit patients with methicillin-resistance staphylococcus aureus: a retrospective cohort study

**DOI:** 10.1186/s12879-025-11510-4

**Published:** 2025-09-16

**Authors:** Heba Swidan, Mayada Salama, Eman Saleh, Ayat Mohamed, Salma Tarek, Rahma Sweedy

**Affiliations:** 1 Al- Gomhoreya General Hospital, Ministry of Health, Alexandria, Egypt; 2 Health Affairs Directorate, Ministry of Health, Dakahlia, Egypt; 3 Health Affairs Directorate, Ministry of Health, Alexandria, Egypt; 4 Research Department, MED-EL, Cairo, Egypt

**Keywords:** Antimicrobial stewardship program, ASP, Defined daily dose, DDD, Linezolid, Timeout process, Methicillin-Resistance staphylococcus aureus, MRSA, Egypt

## Abstract

**Background:**

The empirical use of linezolid as a first-line agent for Methicillin-Resistant Staphylococcus Aureus (MRSA) without a clear indication is of high concern. This study aims to investigate the impact of an Antimicrobial Stewardship Program (ASP) on linezolid consumption and susceptibility in Intensive Care Unit (ICU). In particular, for patients with MRSA infection, ensuring optimized anti-MRSA therapy to align with international/national guidelines.

**Methods:**

A retrospective cohort study was conducted from 01.10.2022 to 31.03.2024 in the ICU of Al-Gomhoreya General Hospital, Alexandria, Egypt. This study included a total of 168 ICU adult patients; older than 18 years old and whom were prescribed anti-MRSA therapy (vancomycin, teicoplanin, or linezolid).

The present study assessed the impact of ASP implementation by comparing six months before ASP, i.e. from 01.10.2022 to 31.03.2023 and following ASP implementation from 01.10.2023 to 31.03.2024. Hence, evaluating adherence to hospital protocol for MRSA management and antibiotic timeout process. One of the principal elements that this study focused on was to quantify the consumption of Linezolid before and after ASP implementation, which was measured utilizing the World Health Organization (WHO) standardized Daily Defined Dose (DDD) per 100 patient days. Thus, enabling evaluating the effect of reducing linezolid usage on MRSA susceptibility to Linezolid and analyzing the overall expenditure on anti-MRSA therapy. The comparative analyses were performed using Permutation Welch Two Sample T-test for the continuous measures, while Chi-squared test or Fisher’s exact test were utilized for categorical outcomes.

**Results:**

Following ASP implementation, it was found that adherence to MRSA indication and timeout process significantly increased by approximately 74.3% and 57.9%, with (*p* values < 0.001 for both), standardized effect sizes (φ) of 0.70 and 0.55, respectively, Linezolid consumption decreased by approximately 85.8% and MRSA sensitivity to Linezolid improved by 18.3%. Furthermore, a reduction of 43% in the overall cost of anti-MRSA therapy was observed.

**Conclusion:**

It was found that implementing an ASP contributes to a substantial reduction in Linezolid consumption and preserving its efficacy by maintaining MRSA susceptibility, while improving adherence to hospital protocols and timeout process. Additionally, it reduces overall expenditures on anti-MRSA therapy. These findings highlight ASP as a viable strategy for combating antibiotic resistance, particularly in resource-limited settings.

## Introduction

Antimicrobial resistance (AMR) arises when pathogens develop resistance to the medications designed to combat them, leading to infections that are much complex and costly be treated. It is among the most challenging global threats today, one of the key challenges is jeopardizing the attainment of Sustainable Development Goals (SDGs) related to health, poverty, food security, and the environment [1].

The Centers for Disease Control and Prevention (CDC) states that AMR is an urgent global public health threat, killing at least 1.27 million people worldwide and associated with nearly 5 million deaths in 2019 [2]. The World Health Assembly urged all countries to develop national action plans by 2017 to combat AMR, in alignment with the global action plan’s goals [3, 4].

In 2017 WHO introduced “WHO AWARE classification” by the WHO Expert Committee on selection and use of Essential Medicines as a tool to support antibiotic stewardship efforts at local, national and global levels. Antibiotics are classified into three groups, Access, Watch and Reserve, taking into account the impact of different antibiotics and antibiotic classes on AMR, to emphasize the importance of their appropriate use [5].

Egypt established its national action plan for 2018–2022, that focused on a multi-sectoral approach to control and combat AMR. ASP implementation in hospitals aim to decrease antimicrobial-resistant strains by improving medication selection, dosing, and duration. Such programs enhance patient safety, reduce morbidity and mortality, and lower the length of hospital stays, thus reducing overall healthcare costs [4, 6].

Data on ASPs in lower-and middle-income countries is limited and required urgent attention [7]. Accordingly, implementing a robust, multidisciplinary ASP, incorporating both pre- and post-prescription strategies in alignment with the WHO toolkit and Infectious Diseases Society of America (IDSA) guidelines, is crucial [8, 9].

Antibiotic consumption is reported to be ten times higher in ICUs compared to other hospital units. Approximately 20–50% of hospitalized patients and 30–60% of ICU patients are prescribed unnecessary, inappropriate, or suboptimal antibiotic treatments [10].

Linezolid, approved by the FDA in 2002, is the first agent in a new class of antibiotics (oxazolidinones) used to treat hospital- and community-acquired pneumonia and complicated skin infections caused by Gram-positive bacteria, including MRSA. Linezolid is considered an alternative to Vancomycin in treating nosocomial pneumonia caused by MRSA. However, misuse of Linezolid can induce resistance, so its prescription should be reserved to severe MRSA infections, especially in cases where Vancomycin is ineffective [11].

In the hospital, the empirical use of linezolid as a first-line agent for MRSA coverage without a well-defined indication, despite its classification as a reserve group antibiotic under the WHO AWaRe classification, in addition to nonadherence to Gram stain results and the failure to perform antibiotic time-out process, contributes to this issue [12].

The prevalence of MRSA and its susceptibility to Vancomycin and Linezolid in Egypt remain under-researched. A study by WHO, indicated that the prevalence of MRSA in Egypt was 46%, and a systematic review found it to be 61% in Alexandria, Egypt. The first meta-analysis 0n MRSA prevalence in Egypt indicated an overall rate of 63%, with resistance rates to Vancomycin and Linezolid of 9% and 5%, respectively [13].

This higher prevalence, compared to other countries, may be attributed to the widespread misuse of antibiotics and self-medication practices, which highlights the urgent need for educational initiatives for healthcare workers on the proper use of antimicrobials [14]. The misuse and extensive use of Linezolid in clinical practices are expected to reduce its efficacy over time, threatening its status as a last-resort antibiotic [15].

Therefore, there is a growing need for efforts focused on the restriction of linezolid use, particularly in light of the existing gap in Egypt and the rising prevalence of MRSA. Our team has initiated work in this area, however, further collaboration is essential to address the issue of the uncontrolled availability of linezolid in community pharmacies, where it is often dispensed without a prescription and used as an empirical treatment.

This study emphasizes the critical role of ASP in tracking MRSA susceptibility to linezolid following its consumption reduction, offering a strategic approach to combat AMR in intensive care units at general hospitals in Egypt [6]. Evaluating the impact of adherence to hospital protocol for MRSA management and timeout process on reducing overall anti-MRSA consumption, particularly linezolid use, is critical.

## Methods

A retrospective cohort observational study was carried out in the ICU department of Al-Gomhoreya General Hospital, Alexandria, Egypt from 01.10.2022 to 31.03. 2024. This study received ethical approval from the Egyptian Institutional Review Board on August 28, 2024. The study aims to evaluate the impact of ASP implementation by comparing a six-month period before ASP implementation from 01.10. 2022 to 31.03. 2023, with a six-month period after implementation from 01.10. 2023 to 31.03.2024, focusing on process and outcome measures; adherence to hospital-specific MRSA management protocol, annual local antibiogram, prior Linezolid authorization, prospective audit, feedback, and antibiotic timeout, collectively enhancing stewardship effectiveness [8, 16]. The total bed capacity was 14. 168 ICU-admitted adults older than 18 and receiving anti-MRSA therapy (vancomycin, teicoplanin or Linezolid) were included in this study. Patients under this age or not using anti-MRSA therapy were excluded. Data was collected from hospital medical records over a six-month period before and after the intervention by clinical pharmacists, with disagreements resolved by consensus. The analysis included patient demographics, consumption of anti-MRSA agents—particularly linezolid—adherence to hospital protocol for MRSA management, and compliance with the time-out process. Additionally, the susceptibility of MRSA to linezolid was assessed using data obtained from the hospital’s annual local antibiogram. The laboratory employed the disk diffusion method (Kirby-Bauer technique) to determine the percentage of MRSA isolates sensitive to linezolid and results were interpreted according to CLSI guidelines (2022), Quality control was conducted using S. aureus ATCC 25,923 as a reference strain, pre-ASP phase encompassed the period from January 1 to December 31, 2023, while the post-ASP phase covered January 1 to December 31, 2024, the percentage of MRSA sensitivity to Linezolid was compared between these two intervals to assess the effectiveness of ASP implementation.

### Pre-ASP

From 01.10. 2022 to 31.03. 2023, the empirical coverage of MRSA in ICU without adherence to hospital-specific MRSA management guidelines, this is identified as the primary contributing factor to the rising prevalence of MRSA in Egypt [13].

Linezolid was administered as a first-line agent for MRSA infection. Linezolid was dispensed after prescription without written justification of the indication of prescription and without adherence to timeout process for reassessment. The extensive overuse of linezolid is a prime driver for the emergency of Linezolid-resistant strains [15]. DDD per 100 patient-days for linezolid, vancomycin, and teicoplanin was calculated based on the total antibiotic consumption during the specified period. The total consumption was divided by the WHO-assigned standard DDD values for each antibiotic (1.2 for linezolid, 2.0 for vancomycin, and 0.4 for teicoplanin), followed by normalization per 100 patient-days. The total number of patient-days during this period was 2,547 [17, 18].

### Interventions (ASP-implementation)

From 01.04. 2023 to 31.09. 2023, the intervention was governed by clinical pharmacist specializing in infectious diseases, utilizing a robust ASP measurement process and outcome assessment in accordance with Center of Prevention and Control core-elements [3]. The interventions were delivered in both formal (monthly meetings) and informal (bedside review) formats. Monthly educational sessions for ICU physicians and staff on rational antimicrobial use, since education is considered one of the most basic and effective tools to influence prescriber behavior [14].

The stewardship committee developed hospital protocol for MRSA management align with IDSA guidelines, WHO recommendations and hospital local antibiogram to measure percentage of adherence to MRSA indication and appropriateness use of Linezolid. Incorporating a simplified format to promote and evaluate adherence to the timeout process, this process is guided by Gram stain results provided by the laboratory microbiologist, aiding physicians in determining the appropriate duration of anti-MRSA therapy [19]. Additionally, the protocol supports adherence to the prospective audit and feedback process [8, 16], daily prospective audit and feedback conducted by the ASP team during ICU rounds.

A standardized prescription form was developed by selecting the approved indication for which Linezolid was prescribed [18], adhering to preauthorization process for Linezolid use, requiring approval by an infectious diseases’ consultant [9]. Regular updates of local antibiograms and treatment guidelines shared with clinical teams. The ASP team was multidisciplinary, comprising an infectious diseases consultant, two clinical pharmacists, a clinical microbiologist, and an ICU physician.

A report on the DDD/100 patient-days for previously mentioned anti-MRSA drugs, as a metric tool standardized by WHO, utilized to assess and compare the consumption before and after ASP implementation [20].

### Post ASP

From 01.10. 2023 to 31.03. 2024, Linezolid was prescribed after pre-authorization form signed by ICU consultant and clinical pharmacist with written justification of the indication of prescription [11]. The susceptibility of MRSA to Linezolid improves following the implementation of a pre-authorization process [13].

Adherence to hospital protocol for MRSA management and time-out process has demonstrated improvements [8]. DDD per 100 patient-days for the previously mentioned anti-MRSA agents was calculated as described above, based on a total of 2,518 patient-days during the post-ASP period.

### Definitions

*DDD*: is the assumed average maintenance dose per day for a drug used for its main indication in adults.

*Standardized antibiotic consumption*: total amount of the antibiotic used, adjusted by the WHO DDD.

*Total patient-days*: is calculated as the number of patients multiplied by the average length of stay.

*Timeout*: A structured reassessment of ongoing antibiotic therapy, this typically occurs after 48 to 72 h after the initiation of antibiotics to evaluate whether continued use is appropriate.

*Prospective audit and feedback*: An antimicrobial stewardship strategy where antibiotic use is reviewed by experts after prescription, clinicians receive feedback and recommendations on optimizing therapy.

*Preauthorization process*: A Core Element in ASP Implementation used to regulate and optimize antimicrobial use, it involves requiring approval before certain antimicrobials can be prescribed or dispensed.

#### Sample size calculation

A retrospective cohort study detected the change in mean linezolid consumption measured by defined daily doses (DDD) pre and post intervention as a primary outcome. Assuming that the mean linezolid DDD/100 patient-days per month was 7.63 ± 4.82 pre-intervention vs. 2.73 ± 3.03 post-intervention with a pooled standard deviation 4.03 from (Papan et al. 2021) [22] estimated from median defined daily doses and interquartile range as demonstrated in (Wan et al. 2014) [23]. For a resulted effect size (Cohen’s d) of 1.22, that calculated from the formula: *Cohen’s d = (M2 - M1) ⁄ Spooled; where: Spooled = √ ((SD*_*1*_^*2*^ *+ SD*_*2*_^*2*^*) ⁄ 2)*, at 5% level of significance to achieve 90% power of the study, the minimum total sample size required was 16 patients/group. After adding a 20% increase to account for the incomplete patients ’records, a total of 40 patients (20 patients per group) were required for the study. Calculated using the “pwr” package in R statistical software version 4.2.2.

#### Statistical analysis

In compliance with the study protocol, all demographic data and clinical characteristics for ICU admitted patients were collected and verified. Tracking patients’ antibiotic consumption during their hospital stay prior to and after ASP implementation was demonstrated accordingly. Descriptive analysis for the collected data was performed as mean, standard deviation (SD), and range for quantitative data. While for qualitative variables, count and percentage were applied instead.

Analytical statistics were applied for patients adhered to the ASP implementation in comparison to those prior to implementation regarding the total antibiotic consumption in grams, adherence to MRSA indication and adherence to timeout process (gram stain results). The comparative analyses were performed using Permutation Welch Two Sample t-test that doesn’t rely on the normality assumptions for the continuous measures, while Chi-squared test or Fisher’s exact test were applied for categorical outcomes according to their assumption’s availability. A two-tailed *p* value ≤ 0.05 was considered the threshold of significance. The standardized effect sizes were estimated using Cohen’s D for the antibiotic’s consumptions; where *Cohen’s D = (M*_*2*_ *- M*_*1*_*) ⁄ Spooled; and Spooled = √ ((SD*_*1*_^*2*^ *+ SD*_*2*_^*2*^*) ⁄ 2)* and the mean square contingency coefficient Phi (φ) applied for chi-squared test; *where* *φ = √(χ²/N)*.

## Results

The study was conducted on 168 ICU-admitted adults, of whom about 64.9% were prior to ASP implementation and about 35.1% following the implementation. Their overall average age was 58.4 ± 16.9 years and both genders were approximately distributed equally all over the study population. Therefore, age and gender showed non-statistically significant differences between the study groups. The overall average body mass index (BMI) of patients was 28.6 ± 4.8 tended to be significantly higher among patients following implementation of ASP with an average BMI of 30.5 ± 6.6 versus 27.5 ± 3.1 among those prior to implementation.

Regarding the patients’ laboratory investigation during their hospital stay, the average highest creatinine level reached was 2.6 ± 2.3 mg/dl, while the average lowest level was 1.7 ± 1.5 mg/dl. Furthermore, the average highest level of platelets was 262.1 ± 123.5 × 1000 per microliter, while the average lowest level was 193.0 ± 92.2 × 1000 per microliter. Patient groups prior to and following ASP implementation did not show any significant differences regarding these investigations as illustrated in more detail in Table 1.

**Table 1:** Demographics and laboratory investigations for patients prior to and following ASP implementation

Patients’ characteristics		Prior to ASP *n* = 109 (64.9%)	Following ASP *n* = 59 (35.1%)	Total *N* = 168 (100%)	*P* value
Age in years	Mean (SD)	60.0 (17.0)	55.3 (16.4)	58.4 (16.9)	0.084
	Min – Max	27.0–94.0	18.0–84.0	18.0–94.0	
Gender	Female	58 (53.2)	27 (45.8)	85 (50.6)	0.447
	Male	51 (46.8)	32 (54.2)	83 (49.4)	
BMI	Mean (SD)	27.5 (3.1)	30.5 (6.6)	28.6 (4.8)	0.001**
	Min – Max	15.6–36.7	18.7–58.5	15.6–58.5	
Highest creatinine level (mg/dl)	Mean (SD)	2.7 (2.5)	2.5 (2.0)	2.6 (2.3)	0.464
	Min – Max	0.8–9.2	0.7–8.2	0.7–9.2	
Lowest creatinine level (mg/dl)	Mean (SD)	1.8 (1.5)	1.6 (1.5)	1.7 (1.5)	0.482
	Min – Max	0.7–5.8	0.4–6.4	0.4–6.4	
Highest platelets level (× 1000/mcL)	Mean (SD)	253.9 (89.2)	277.5 (169.6)	262.1 (123.5)	0.332
	Min – Max	95.0–450.0	50.0–999.0	50.0–999.0	
Lowest platelets level (× 1000/mcL)	Mean (SD)	189.9 (67.9)	198.8 (125.8)	193.0 (92.2)	0.607
	Min – Max	75.0–400.0	21.0–589.0	21.0–589.0	

^**^0.01 >*p* value ≥ 0.001

### Comparative analyses of clinical outcomes represented in Table 2

**Fig. 1:**
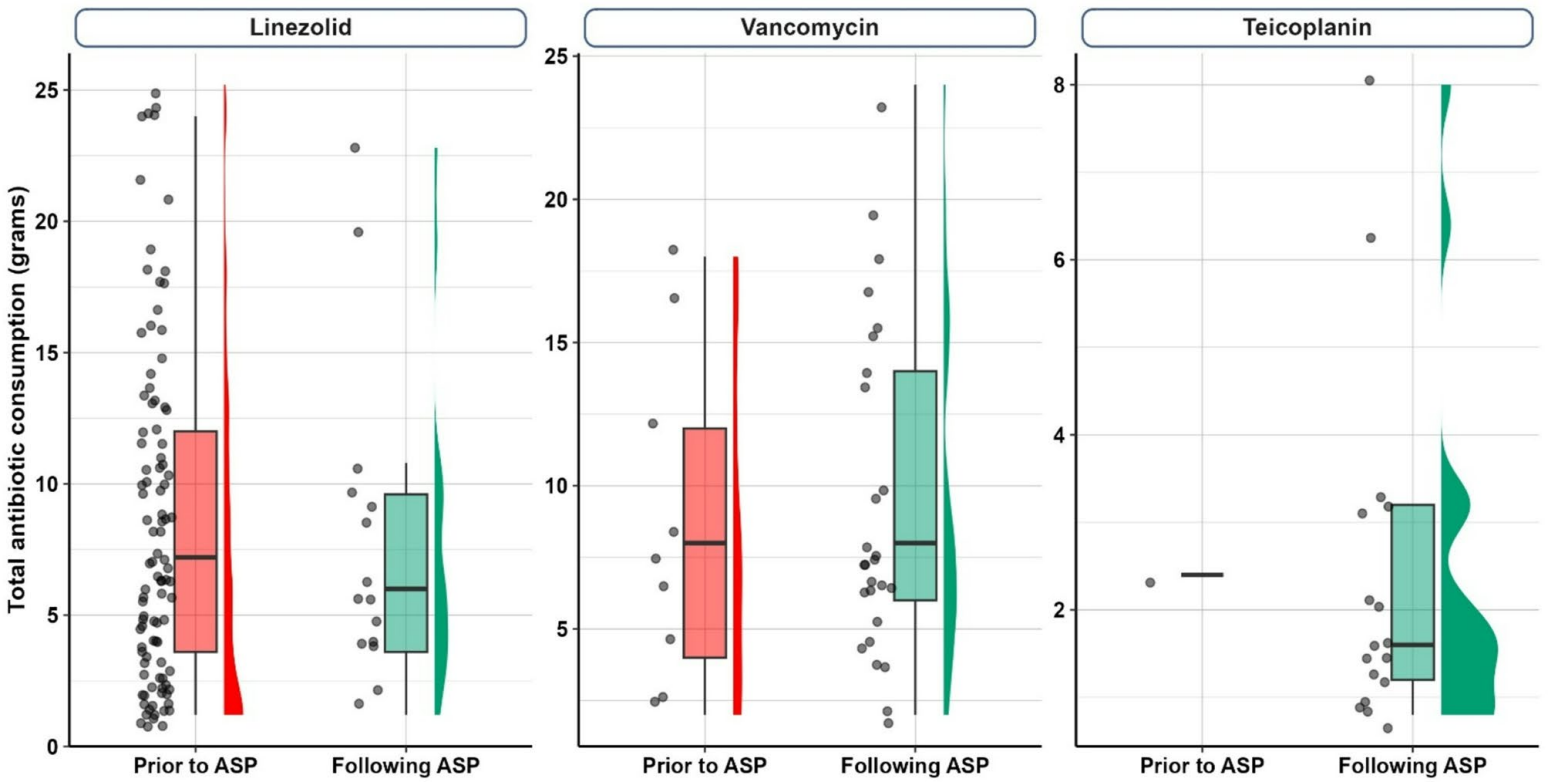
Total antibiotic consumption in grams prior to and following ASP implementation for Linezolid, Vancomycin, and Teicoplanin

Regarding antibiotic consumption, patients following ASP implementation exhibited an observed non-significant decrease in the total Linezolid consumption from an average of 8.4 ± 6.4 gm prior to implementation to 7.8 ± 6.1 gm post implementation with a standardized effect size (Cohen’s d) of 0.10 with 95%CI: (−0.45, 0.64) representing about 7.1% decreased percentage in total Linezolid consumption. However, an observed non-significant increase in the total Vancomycin consumption from an average of 8.4 ± 5.8 gm prior to ASP implementation to 9.4 ± 5.8 gm post implementation with a standardized effect size (Cohen’s d) of 0.17 with 95%CI: (−0.92, 0.59) representing about 11.9% increased percentage in total Vancomycin consumption. Teicoplanin administration also increased following the ASP implementation since only one patient had received it prior to implementation consuming only 2.4 gm while the average total consumption post implementation reached 2.4 ± 2.0 gm with a standardized effect size (Cohen’s d) of 0.02 with 95%CI: (−1.99, 2.04) as demonstrated in Fig. 1.

**Fig. 2:**
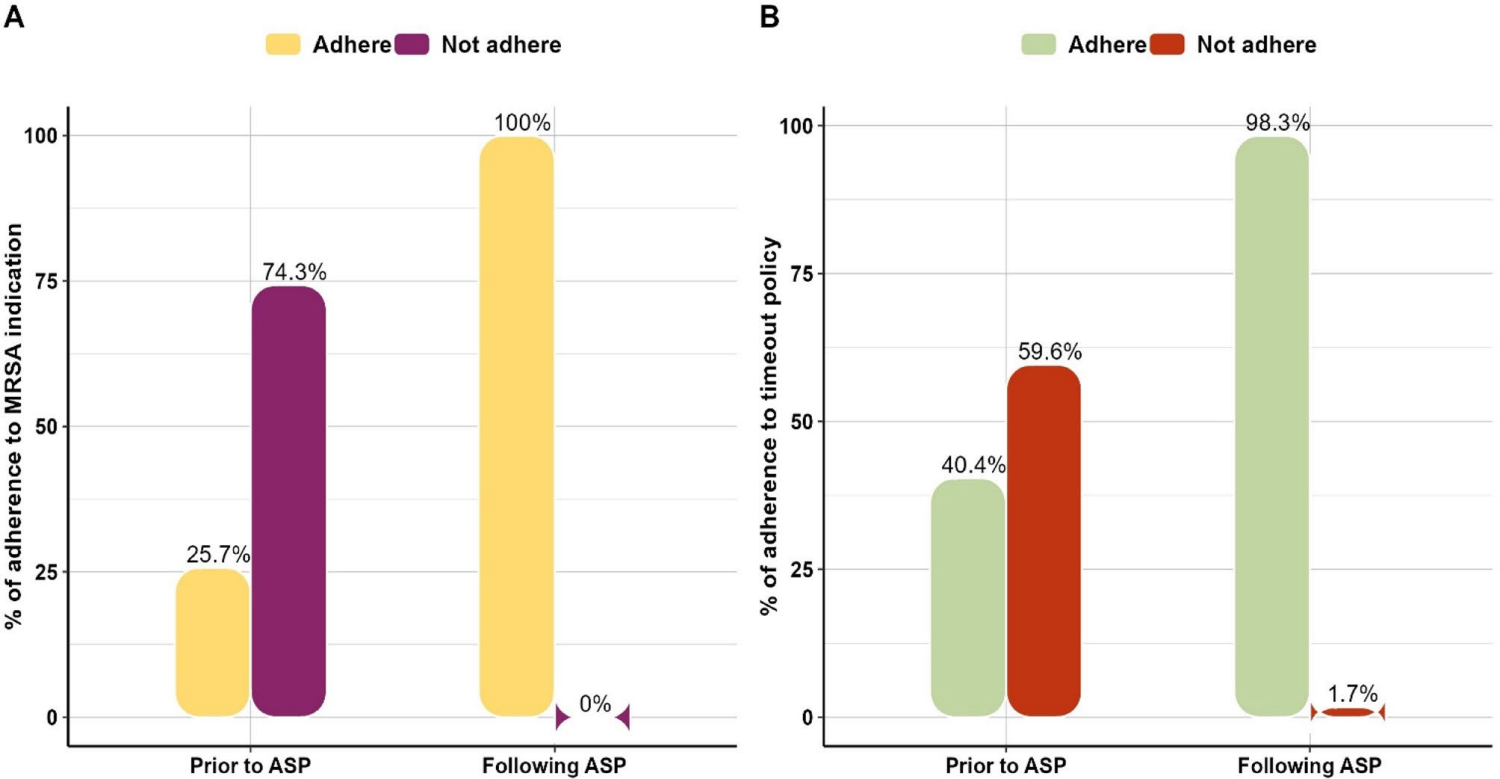
Comparative analyses for patients prior to versus following ASP implementation regarding; (**A**): Adherence to MRSA indication, and (**B**): Adherence to timeout policy (gram stain results)

Regarding the percentage of adherence to MRSA indication, 100% of patients following ASP implementation were adhering to MRSA indication compared to only 25.7% adherence among those prior to implementation. About 74.3% increase in the adherence percentage to MRSA indication was observed post implementation showing a statistically significant improvement (*p* < 0.001) with a large, standardized effect size (φ) of 0.70 with 95%CI: (0.65–1.07) **(**Fig. 2-A**)**.

Moreover, the adherence to timeout policy, considering gram stain results, increased significantly from 40.4% among patients prior to the ASP implementation to 98.3% following the implementation (*p* < 0.001). The percentage of increase in adherence to timeout policy was about 57.9% with a large, standardized effect size (φ) of 0.55 with 95%CI: (0.44–0.80) **(**Fig. 2-B**)**.

Table 2 showed the calculated DDD per 100 Patient-Days for each antibiotic indicating that:

**Table 2:** Comparative analyses between patients prior to and following ASP implementation regarding antibiotic consumption, adherence to MRSA indication, adherence to timeout policy (gram stain results), DDD per 100 Patient-Days for each antibiotic use, overall cost in EGP and MRSA sensitivity to linezolid

Patients’ outcomes		Prior to ASP	Following ASP	Effect size (95%CI:)	*P* value
• Total antibiotic consumption in grams:					
Linezolid	Mean (SD)	8.4 (6.4)	7.8 (6.1)	0.10 (−0.45, 0.64) ^**a**^	0.721
	Min – Max	1.2–25.2	1.2–22.8		
Vancomycin	Mean (SD)	8.4 (5.8)	9.4 (5.8)	0.17 (−0.92, 0.59) ^**a**^	0.675
	Min – Max	2–18	2–24		
Teicoplanin	Mean (SD)	2.4 (0.0)	2.4 (2.0)	0.02 (−1.99, 2.04) ^**a**^	--
	Min – Max	2.4–2.4	0.8–8.0		
• Adherence (%):					
Adherence to MRSA indication, n (%)	Adhere	28 (25.7)	59 (100.0)	0.70 (0.65, 1.07) ^**b**^	< 0.001***
	Not adhere	81 (74.3)	0 (0.0)		
Adherence to timeout policy (gram stain results), n (%)	Adhere	44 (40.4)	58 (98.3)	0.55 (0.44, 0.80) ^**b**^	< 0.001***
	Not adhere	65 (59.6)	1 (1.7)		
• DDD per 100 Patient-Days for each antibiotic use:				**Amount of change**	
Linezolid	Overall	27.365	3.891	− 85.8%	--
Vancomycin	Overall	1.491	5.044	+ 2.4 folds	--
Teicoplanin	Overall	0.235	3.970	+ 15.9 folds	--
• Cost:					
overall cost of anti-MRSA therapy (EGP)	Overall	80097.17	45605.27	−43%	--
• MRSA sensitivity:					
MRSA sensitivity to Linezolid	Overall	60%	78.3%	+ 18.30%	--

^***^p value < 0.001, *CI* Confidence Interval, ^a^Cohen’s D effect size, and ^b^Mean square contingency coefficient (φ)

**Fig. 3:**
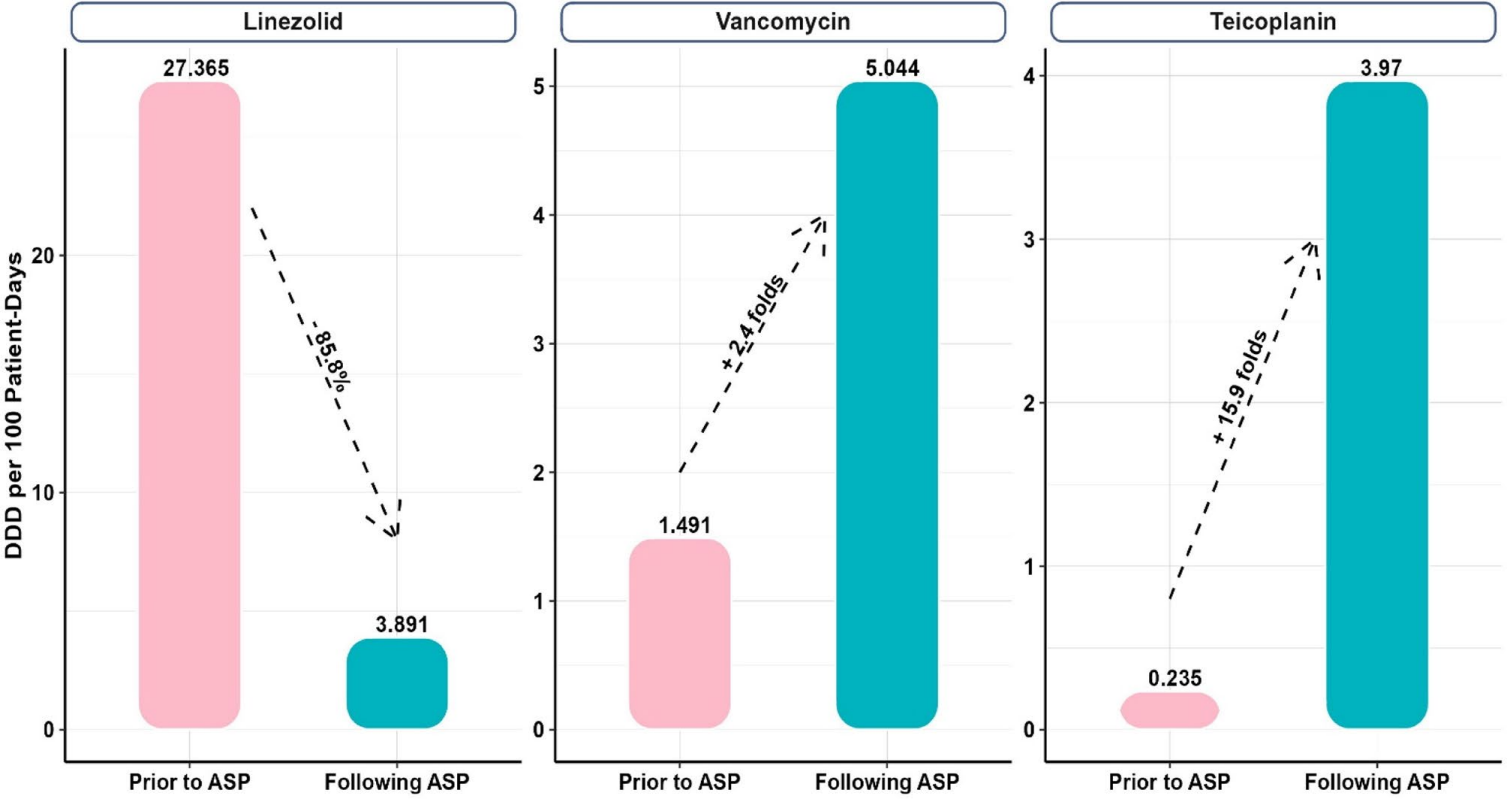
DDD per 100 Patient-Days prior to ASP implementation versus following implementation for Linezolid, Vancomycin, and Teicoplanin

DDD for Linezolid decreased from 27.365 prior to ASP implementation to 3.891 following implementation with approximately 85.8% decrease. However, DDD for Vancomycin increased from 1.491 prior to ASP implementation to 5.044 following implementation exhibiting about 2.4 folds increase. Furthermore, DDD for Teicoplanin showed an observed increase from 0.235 prior to ASP implementation to 3.970 following implementation with approximately 15.9 folds increase as illustrated in Fig. 3.

**Fig. 4:**
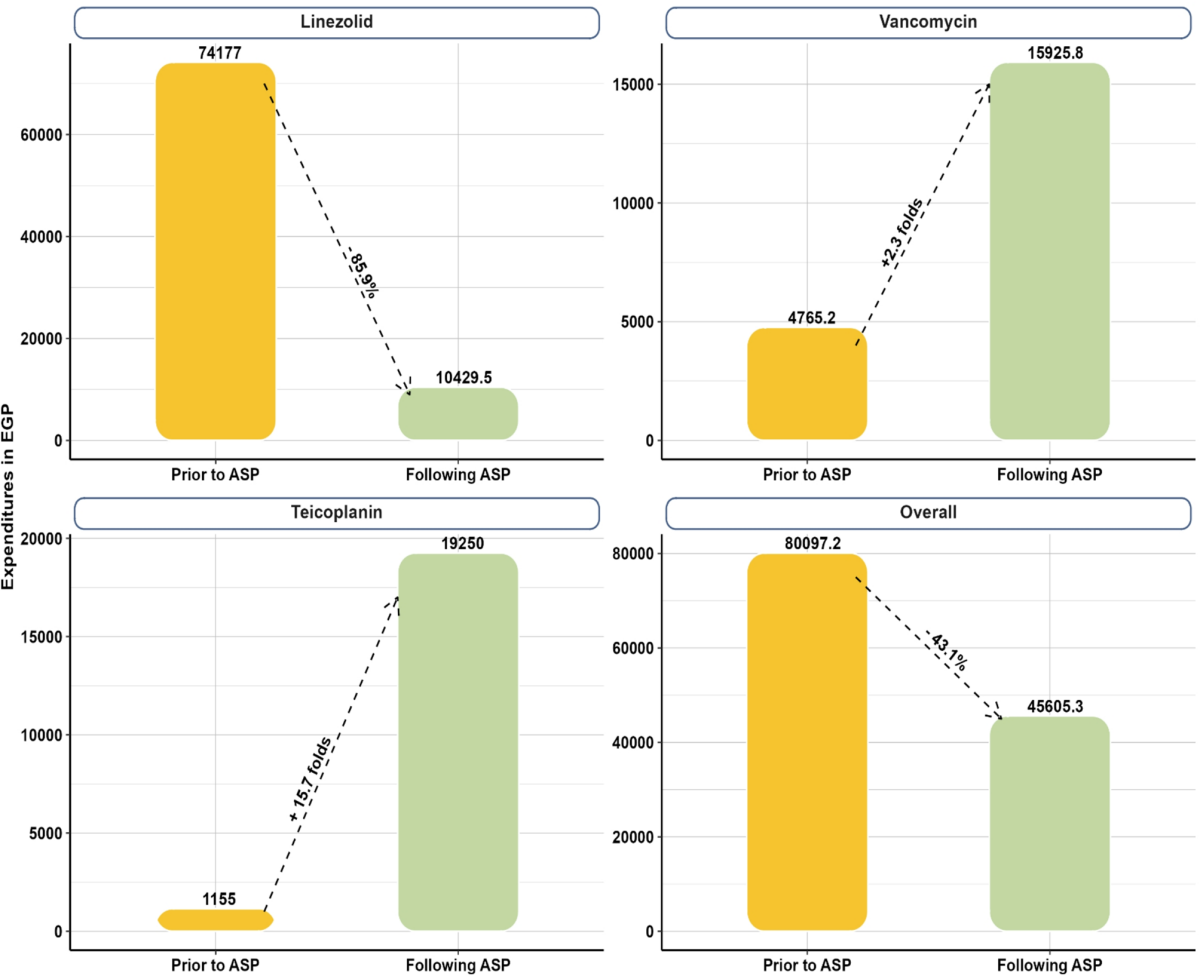
Expenditures prior to ASP implementation versus following implementation for Linezolid, Van*c*omycin, Teicoplanin, and Overall

**Fig. 5:**
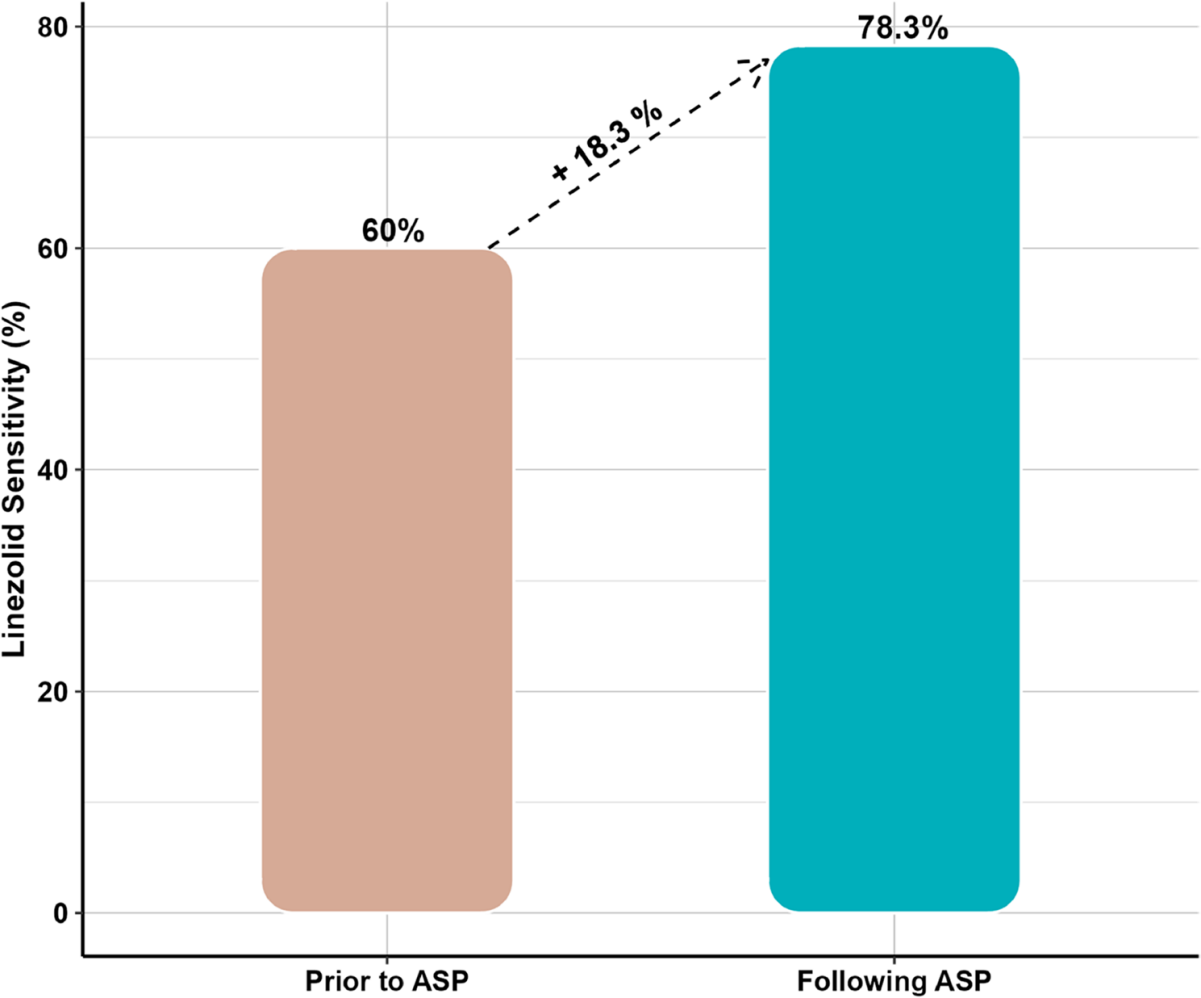
Improvement in Linezolid sensitivity following ASP implementation compared to prior to implementation

Figure 4 illustrates the change in total expenditures, in Egyptian pounds, after ASP implementation where Linezolid showed about 85.9% cost reduction, while expenditures of Vancomycin and Teicoplanin increased by 2.3 folds and 15.7 folds, respectively. However, the overall expenditure decreased following ASP implementation by about 43.1% when compared to prior implementation. Furthermore, the annual local antibiogram detected an observed improvement in sensitivity of MRSA isolates to Linezolid by approximately 18.3% (from 60% prior to ASP implementation to 78.3% following implementation) as shown in Fig. 5.

## Discussion

Our ASP is the first in Egypt to achieve rapid intervention for linezolid misuse while effectively tracking MRSA sensitivity shifts in direct relation to linezolid stewardship.

The implementation of multidisciplinary ASP incorporating both pre-and post-prescription activities in an Egyptian ICU significantly improved adherence to MRSA indication criteria. It also enhanced the enforcement of a timeout policy and the application of a preauthorization restriction form for linezolid prior to prescription, resulting in a marked reduction in linezolid utilization [8]. This, in turn, was associated with enhanced MRSA susceptibility to linezolid and an increased reliance on alternative anti-MRSA agents, such as vancomycin and teicoplanin. Furthermore, these interventions contributed to a reduction in the overall cost of anti-MRSA pharmacotherapy. These findings align with global evidence on ASP effectiveness while highlighting unique challenges and opportunities in the Egyptian healthcare context.

The substantial reduction in linezolid consumption, quantified as decrease of 85.8% in DDD per 100 patient-days following ASP implementation, was subsequently associated with an 18.7% increase in MRSA susceptibility to linezolid. This trend likely reflects the effectiveness of targeted ASP interventions to curb overuse of this last-resort antibiotic, which is critical given its unique mechanism and previous reports of emerging resistance in Egypt [6]. Guillard et al. (2014) demonstrated in their study that an ASP with interventions like education, preauthorization, and ID physician review led to a reduction in linezolid prescriptions [11]. Similarly, Papan et al. (2021) found that unrestricted linezolid use (> 13 DDD/100 patient-days) led to Linezolid-Resistance Staphylococcus Epididymis (LRSE) outbreaks, which were controlled by restricting linezolid [22]. Overuse of linezolid has been linked to the emergence of linezolid-resistant strains, particularly in hospital settings such as ICUs. Hamdy et al. (2022) reported that only 61.8% of Egyptian hospitals had an active ASP that include multidisciplinary teams, which is consistent with international standards, with 65.7% performing susceptibility testing, but 51.4% lacked a formal antibiogram [6]. The assessment of MRSA susceptibility to linezolid in this study was based on microbiological surveillance conducted during the pre- and post-ASP implementation periods. The decrease in linezolid use, therefore, aligns with stewardship goals to preserve its efficacy. The PROVAUR stewardship program highlighted those structured interventions reduced linezolid-resistant CoNS and Enterococcus faecalis resistance by 63% and 56%, respectively, through optimized prescribing [24]. Similarly, linezolid utilization in a German ICU dropped from 7.5 to 2.5 DDD/100 patient-days post-intervention, alongside a significant decline in LRSE prevalence [22]. These results underscore that restricting linezolid through stewardship can reverse resistance trends, even in settings with high baseline usage. Conversely, vancomycin and teicoplanin consumption increased post-implementation, reflecting a shift in prescribing practices. Papan et al. (2021), specifically mentioned promoting vancomycin as a first-line antibiotic for presumed Gram-positive, beta-lactam-resistant bacteria. This suggests a deliberate shift in prescribing patterns facilitated by the ASP [22], conform with cited results DDD per 100 patients for vancomycin and teicoplanin increased by about 2.4 and 15.9 folds respectively following ASP.

Furthermore, adherence to MRSA indication increased from 25.7 to 100% (*p* < 0.001), these findings suggest that ASPs enforcing protocol adherence enhance documentation rigor, particularly for high-risk pathogens like MRSA. Egypt’s National AMR Action Plan (2017–2020), developed with WHO support, further institutionalized surveillance and stewardship, creating a policy backbone for such improvements [4].

While adherence to antibiotic timeout process improved from 40.4 to 98.3% (*p* < 0.001), demonstrating the program’s effectiveness in optimizing antibiotic use. The time-out process enhances appropriate use by ensuring that antibiotic choices remain optimal based on clinical progress and microbiological data [22, 25].This process likely contributed to the observed decrease in linezolid use and the rationalization of anti-MRSA therapy [15]. This be consistent with Papan et al. (2021), who found that antibiotic time-outs combined with ASP interventions reduced LRSE outbreaks [22]. The improvement in time-out compliance in Egyptian hospitals mirrors findings from a U.S. study where mandatory antibiotic time-outs reduced unnecessary therapy by 35% (Baur et al., 2017) [26].

Adherence to a multidisciplinary ASP was associated with a significant reduction in overall antibiotic consumption, resulting in a 43% decrease in the total cost of the previously mentioned anti-MRSA therapy. This finding matches the systematic review by Dij et al. (2000–2014), which reported that 92% of the included studies demonstrated a reduction in antibiotic expenditures. The cost savings were particularly substantial in hospitals implementing comprehensive ASPs that incorporated both therapeutic review and antibiotic restriction strategies [7].

The study emphasizes the importance of ASP’s role in improving antibiotic prescribing behaviors, reducing unnecessary linezolid use, and potentially preserving MRSA sensitivity to Linezolid, stressing the requirement for wider adoption of stewardship strategies. Moreover, the uncontrolled availability of linezolid in community pharmacies, where it can be dispensed without a prescription as an over-the-counter (OTC) medication, poses a significant threat to its efficacy and contributes to antimicrobial resistance. We recommend Immediate regulatory measures should be implemented to restrict its distribution, ensuring it is available exclusively in hospital pharmacies and used under strict medical supervision to preserve its therapeutic effectiveness.

### Limitations

Our study has certain limitations. First, our findings do not comprehensively represent all hospital departments, as our focus was primarily on the ICU—the department with the highest recorded consumption of anti-MRSA drugs. To maximize the impact of our ASP, it must be expanded to encompass all hospital departments. Furthermore, for broader effectiveness, our ASP should be implemented across a wider range of hospitals in Egypt, ensuring a more substantial and sustainable impact on antimicrobial resistance management.

Second, the inclusion of fewer than 30 MRSA isolates in the annual local antibiogram, which may compromise accuracy according to Clinical and Laboratory Standard Institute (CLSI)guidelines [27]. To address this limitation, it was previously noted that collecting 30 isolates may not be feasible within a short time frame, especially in smaller institutions or for rare pathogens, therefore, even if the isolate count is low, this helps in tracking resistance trends and guiding empirical therapy.ASP often accept smaller numbers of isolates for internal tracking or alerts, especially for emerging resistance [28].

Additionally, we advocate for further research to elucidate the relationship between linezolid stewardship, microbial sensitivity, and clinical efficacy. Such investigations are essential to ensure the optimal use of linezolid and support its long-term preservation.

Third, as this was a retrospective study, essential clinical variables such as APACHE II scores, SOFA scores, and comorbidity data were not consistently available, limiting our ability to adjust for potential confounders, additionally, there is a possibility of information bias. Future prospective studies with more comprehensive data collection may help mitigate these limitations. Fourth, due to resource limitations, genotypic confirmation of linezolid-resistant Staphylococcus aureus isolates—specifically testing for cfr, optrA, or poxtA resistance genes—was not performed. As a result, the precise genetic mechanisms underlying any observed phenotypic resistance could not be determined, which may limit the microbiological depth of our findings.

We also recommend future studies monitor the sensitivity of MRSA to vancomycin and teicoplanin as potential alternative anti-MRSA agents following the restriction of linezolid use. Despite these limitations, this study successfully identified practical strategies to preserve one of the WHO AWaRe-classified “Reserve” group antibiotics and mitigate resistance to this last-line treatment in intensive care units.

## Conclusion

Addressing antibiotic misuse in resource-limited settings requires a pragmatic approach. Implementing ASP tailored to align with global best practices offers a pioneering strategy to combat antibiotic resistance and safeguard the efficacy of threatened antimicrobial agents in developed countries. The implementation of a well-structured, criteria-based ASP by a highly educated and self-committed team can lead to significant success and impactful outcomes. While achieving success is commendable, sustaining and continuously enhancing the program is the ultimate goal deserving recognition.

## Data Availability

All data generated or analyzed during this study are included in this publishedarticle and its related files. Further details are available from the corresponding author upon reasonable request.

## Abbreviations

ASP: Antimicrobial Stewardship Program
MRSA: Methicillin-resistant Staphylococcus Aureus
CI: Confidence interval
WHO: World Health Organization
ICU: Intensive Care Unit
AMR: Antimicrobial resistance
DDD: Daily Defined Dose
CDC: Centers for Disease Control and Prevention
IDSA: Infectious Diseases Society of America
SD: Standard deviation
LRSE: Linezolid-resistant Staphylococcus epidermidis
CLSI: Clinical and Laboratory Standard Institute
EGP: Egyptian pounds
